# The Shortage in Hospital Medical Staffs: The Facts and the Remedies

**Published:** 1915-06-05

**Authors:** 


### THE SHORTAGE IN HOSPITAL MEDICAL STAFFS.

### The Facts and the Remedies.

The voluntary hospitals throughout the country, with zeal, efficiency, and self-denial, have rendered vast services to the Government and the Empire. They have unselfishly made provision for the medical and surgical treatment of the sick and wounded in their hospitals and throughout the Services. They have further been prodigal in their generous acceptance of war conditions whereby the numbers of their medical and surgical staffs have been continuously diminished until some of the larger hospitals, with numerous smaller ones, are face to face with the difficulty of deciding how to carry on the work of the hospital from day to day. The problem involves visiting physicians and surgeons, members of all branches of the resident medical staff, students who hold appointments as dressers, nurses, and even members of the civil staff. From the outset of the war the present shortage has been foreseen; and it has for some time past entailed heavy responsibilities on the administration and much self-denial on the part of the workers in most branches of the hospital service. There are instances of members of the honorary staff, whenever a new batch of wounded has been received from the Front, continuing at work in the hospital, rendering every sort of assistance in addition to their surgical work, and remaining on duty until the early hours.

It is well that these facts should be apprehended by the people throughout the United Kingdom. The voluntary hospitals want special pecuniary help in liberal measure, and will want it more every month as the war proceeds. These facts should be welcomed by Dives and by all who are able to give something to the hospitals for the sick and wounded. Some of the noblest types of men we have met have been men of wealth or means who have been liberal givers when the need was urgent and the cause noble. Whilst the majority of people who have substance are keenly anxious to do their part at this time of national crisis, it is now that the more intelligent amongst them at any rate should awaken to the work, the claims, and the needs of the voluntary hospitals, and make it their business to supply them with funds in ample measure.

Let us examine this question of shortage so far as it affects the honorary and resident medical staff of the hospitals. The reception of sick and wounded means for each hospital staff much additional pressure on their time and services. It has been the aim of the managers of voluntary hospitals admitting sick and wounded sailors and soldiers to provide for them without in any way diminishing the supply of a full complement of beds and treatment for the civil population. In order to secure this result it has been necessary to increase the number of beds in certain of the larger wards, such increase amounting to 33 per cent., which is the absolute maximum if hygienic laws are to be enforced. It has been a great tax on the powers of the honorary and resident staff of a voluntary hospital to complete efficiently each day's work when so extended; but this burden has been and is being continuously added to by the desire of the younger men to enter the Royal Army Medical Corps, and by the spirit which animates every worthy inhabitant of the United Kingdom and Empire to take some part in their active defence. The members of the medical profession attached to hospitals, both young and old, have rendered splendid service which does them honour; but they cannot do impossibilities. The position at the moment in some hospitals is that unless new measures are taken to meet the shortage in the medical staff it will be impossible to supply a succession of men to take up resident appointments.

Every care has been taken to utilise and economise the existing service, and nothing further in this direction can be accomplished. In the case of one great hospital, by the middle of June, as matters stand at present, the resident appointments will be vacated, and there will be practically no applicants to replace the present men. The work to be done is increasing. Including the extra beds for the wounded, the total reaches about 1,200. This hospital is under further obligation to provide the Admiralty, in accordance with their promise, with 250 additional beds, should they be called for. Wounded arrive at the hospital almost daily, and already 3,000 soldiers from the war have been under treatment there. In addition to this, the work imposed upon the casualty and out-patient departments is continuously heavy. The honorary medical staff are working like Trojans. They continuously pay extra visits, and several of them have expressed a willingness to undertake the duty of a resident in turn. Generous as these offers are, they cannot, however, supply a number of house physicians and house surgeons whose continuous presence is essential for efficient work.

At another great hospital in London the same difficulties have had to be met, but in this case it is attached to an old medical school, which gives it the advantage of having a great number of old students engaged in practice, or retired from practice, who have, almost to a man, offered their services. The shortage difficulty has increased continuously since August, and it has nearly reached its climax at the present time owing to the marked effect which the appeal for recruits has had upon the rising generation of students and junior medical officers. This is so keen that students have left the hospitals in a body for the Front, many of them joining as privates. In the same way several members of the honorary medical staff have been or are at present at the Front. The great difficulty is the shortage in resident medical officers, and this difficulty must be promptly met and overcome. Further, the departure of the students has left the hospital with too few dressers, and substitutes must be provided.

Let us next consider what steps can be taken to overcome the difficulties of the present situation in certain large voluntary hospitals. Taking first the question of dressers, we may point out that the R.A.M.C. orderlies who have been recruited for training amount to some thousands, as we are advised, and that at the present time opportunities to train them in handling and nursing the sick are not available in the camps where they are undergoing a military training. Here, then, is clearly a case where an arrangement should be come to between the War Office and the civil hospitals whereby both would immediately benefit. Already at the London Hospital orderlies are sent there for a few months' training. These men are distributed through the wards, operation theatres, casualty and out-patient departments. They sleep on mattresses on the floor in the out-patient department, and the War Office allows the hospital 1s. 9d. a day for the food of each orderly. Each batch is sent up in charge of a qualified R.A.M.C. lieutenant. The report from the London Hospital is that it would be difficult to exaggerate the usefulness of these men. They have overcome a grave difficulty, and the House Governor would warmly recommend other hospitals to approach the War Office for relays of orderlies on a similar plan. So far as the orderlies are concerned, the benefits which they have received in practical training can hardly be exaggerated. Each orderly is a picked man, and spreads the knowledge he gains in the voluntary hospital to the rest of his party when he gets back to his military camp. The orderlies are always in attendance on the admission of wounded during the day or night, and thus gain a very practical experience in the handling of stretchers and in the conveyance of dangerously wounded men. These facts are well worth the consideration of those whom they concern.

In regard to the shortage of practitioners two methods have been suggested. The first is that followed at St. Thomas's Hospital, where an appeal has been made to the whole body of old students which has resulted in largely overcoming the difficulties which we have explained, owing to the patriotic way in which old students have come forward and by personal sacrifice arranged to supply the deficiency of workers throughout the medical and surgical sides of the hospital. In the case of other hospitals with large medical schools no doubt a similar plan has been or will be followed, but where circumstances prevent an adequate supply of practitioners from these sources other steps must be taken. In considering these steps it is well to remember that men who have just gained the qualification at the quarterly Conjoint examinations are not in the majority of cases adequately equipped for the medical service required at the Front. The same objection applies to many members of the profession who have been in private practice and long absent from the hospitals. Both types would be immeasurably benefited and would have an adequate chance of proving useful and making progress at the Front could they, before joining the Colours, receive three months' practical and responsible experience in a great hospital. Why should not arrangements be made by the War Office to utilise and at the same time to help our great medical institutions at home by entering into an agreement with such institutions to send the newly qualified men and a selected number of the private practitioners who apply for commissions to one of the larger hospitals for this practical and responsible experience?

### An Admirable Scheme.

An arrangement of this kind is one which would adapt itself efficiently to meet the ever-pressing claims of a great war which cannot in all probability be a short one. It would admirably fit in with and fulfil the objects to be met by mobilising the nation. It is a practical way of placing national service, so far as the medical profession is concerned, upon a business basis; and it must result not only in benefit to the medical service throughout our armies, but the after-effects of such a system must later on prove highly beneficial to the interests of the civil population. An admirable scheme to carry out the plan just indicated has been prepared by certain practical hospital men who know all the circumstances, and by their wide knowledge of the facts are entitled to be consulted. One great obstacle to the speedy organisation with the maximum efficiency of British enterprise, not only in Government but in civil life, is the habit of following an established routine, and the difficulty of breaking through the entanglements incidental to a system which must be modified to the verge of annihilation, if this country is ever adequately to hold its own with other nations throughout the world.

Finally, there is one other fact in this connection which it may be useful for the hospitals which are suffering from shortage to know. A considerable number of medical men of all ages, qualifications, and experience have sent in their names to the War Office offering service in the existing emergency. Some can give two half-days a week, some can give more, some can act as anæsthetists, some as supplementary honorary officers, some as temporary residents, and so forth. A list, we understand, of these offers has been made at the War Office; but to bring it into full use and to make its value as great as possible it is essential that that list should be made available for the information of all the hospitals which have a shortage in their medical service. We have heard men who have sent in their names complain that willing and anxious as they are to render medical aid, their services, if they may judge from the want of response their applications have received, are not required. This is not the case. The impression has got abroad because the list has not been made generally available. We hope that the War Office may consent to have this list made immediately effective by inviting all institutions where there is a shortage to apply to the War Office, so that each of them may select from the list such offers of medical service as may be best adapted to fulfil the requirements of every voluntary hospital.

